# Antiretroviral Therapy Use Was Not Associated with Stillbirth or Preterm Birth in an Analysis of U.S. Medicaid Pregnancies to Persons with HIV

**DOI:** 10.1089/whr.2023.0040

**Published:** 2023-08-25

**Authors:** Kathryn D. Thompson, David J. Meyers, Yoojin Lee, Susan Cu-Uvin, Angela M. Bengtson, Ira B. Wilson

**Affiliations:** ^1^ Department of Health Services, Policy and Practice, Brown University School of Public Health, Providence, Rhode Island, USA.; ^2^ Warren Alpert School of Medicine, Brown University, Providence, Rhode Island, USA.; ^3^ Providence/Boston Center for AIDS Research (CFAR), Brown University, Providence, Rhode Island, USA.; ^4^ Department of Epidemiology, Brown University School of Public Health, Providence, Rhode Island, USA.

**Keywords:** ART, birth outcomes, preterm birth, stillbirth, Medicaid

## Abstract

**Background:**

Using a U.S. based, nationally representative sample, this study compares stillbirth and preterm birth outcomes between women living with HIV (WWH) who did and did not use antiretroviral therapy (ART) during pregnancy, additionally assessing ART duration and regimen type.

**Methods:**

Using 2001 to 2012 Medicaid Analytic eXtract (MAX) data from the 14 states with the highest prevalence of HIV. We estimated two, propensity score matched, multivariate logistic regression models for both outcomes of stillbirth and preterm birth: (1) any ART use and (2) the number of months on ART during pregnancy for ART users, adjusting for patient-level covariates.

**Results:**

Only 34.6% of pregnancies among WWH had a history of ART use and among those, the proportions of stillbirth and preterm birth were 0.9% and 7.9%, respectively. Any ART use was not significantly associated with either outcome of stillbirth (marginal effects [MEs]: 0.06%, 95% confidence interval [CI]: −0.17 to 0.28) or preterm birth (ME: −0.12%, 95% CI: −0.79 to 0.55). For ART users, duration of ART was not significantly associated with either outcome. Black race was a strong independent predictor in both models (stillbirth: 0.80% and 0.84%, preterm birth: 4.19% and 3.76%). Neither protease inhibitor (PI) nor boosted PI regimens were more strongly associated with stillbirth or preterm birth than nucleoside reverse transcriptase inhibitor-based regimens.

**Conclusion:**

ART use during pregnancy was low during this period. Our findings suggest that ART use and ART regimen are not associated, positively or negatively, with stillbirth or preterm birth for mothers with Medicaid. Additionally, our findings highlight a persisting need to address disparities in these outcomes for Black women.

## Introduction

In the United States, nearly 5,000 women living with HIV (WWH) give birth every year.^1^ For WWH, the use of combination antiretroviral therapy (ART) during pregnancy has been recommended since 1994.^2^ This has reduced mother-to-child transmission in the United States to less than 1%,^3,4^ as observed in other parts of the world.^5–7^ ART has clear benefits for maternal health and the health of children.^8,9^

The impact of ART on adverse birth outcomes—such as low birthweight, premature birth, and stillbirth—is more controversial. Some studies from the United States have suggested that ART is not associated with adverse birth outcomes,^10–14^ but other U.S. studies have suggested that ART, and protease inhibitor (PI)-based ART in particular, may be associated with adverse outcomes.^15–17^ The published literature on the impact of ART on birth outcomes is complicated by differences in populations, study designs, and the ability to adjust for confounding. Also, over time there have been changes in the indications for who received different regimens, producing confounding by indication or channeling bias.^9^

Most of the data available to address the issue of whether ART is associated with adverse birth outcomes in the United States come from cohort studies or clinical trials. Population-based studies, if they are from the modern ART era, can usefully supplement these studies, particularly if they compare ART users to nonusers. We recently showed, in a large, nationally representative sample of pregnant women covered by Medicaid, that rates of stillbirth and prematurity were similar in WWH and HIV-negative women.^18^

Utilizing a U.S. based, nationally representative sample, in this article we build on our prior work with U.S. Medicaid data to assess associations of ART with stillbirth and preterm birth in over 30,000 deliveries for WWH that took place between 2001 and 2012. We had three main questions. First, is ART use, compared to no use, positively or negatively associated birth outcomes (*i.e*., stillbirth and preterm birth)? Second, is there a relationship between the number of months of ART during the year before delivery and adverse birth outcomes (*i.e*., stillbirth and preterm birth)? Third, is ART regimen type associated with stillbirth and preterm birth outcomes?

## Methods

### Data

We used the Centers for Medicare and Medicaid Services (CMS) Medicaid Analytic eXtract (MAX) administrative claims from January 1, 2001, to December 31, 2012. Medicaid is a public, state, and federal insurance program and covers nearly 40% of individuals living with HIV in the United States. MAX data include the Annual Demographic and Eligibility File, and four claim type files: Inpatient, Long Term Care, Pharmacy, and Other Therapy. We used Medicaid claims from the 14 states with the highest HIV prevalence: California, Florida, Georgia, Illinois, Louisiana, Massachusetts, Maryland, North Carolina, New Jersey, New York, Ohio, Pennsylvania, Texas, and Virginia; accounting for nearly 75% of prevalent U.S. HIV infections. We used 2010 Census to identify geographic region and determine rural residence at the county level.

### Study design and study population

This retrospective cohort study used Inpatient Hospital claims within the MAX files that indicated a live- or stillbirth to a WWH. In the study, we refer to individuals denoted as females in the dataset as “women,” recognizing that not all persons who deliver identify as a woman. We included women who were and were not continuously enrolled in Medicaid, as eligibility requirements for Medicaid vary by state. We classified WWH in our sample if (1) there were two or more HIV diagnosis claims at least 30 days apart (International Classification of Diseases 9th Revision Code: 042, V08), or (2) if they used any combination of ART medications during the study period, as ARTs are seldom prescribed for patients without HIV. This approach has been used in previously published studies.^19,20^ Sensitivity analyses were conducted excluding pregnant WWH because of ART use (13.4%). The results of these analyses were similar to the main analyses.

Reasons for exclusion included the following: 7,663,758 were to HIV-negative women, 72,454 had International Classification of Diseases, Ninth Revision, Clinical Modification (ICD-9-CM) coding errors, 6,434 did not specify a birth outcome, 3,450 were coded as males, 19,370 had inadequate information to determine HIV status. We also excluded deliveries of women <18 years of age, complexities related to parental consent for treatment for minors, and those diagnosed with HIV after the delivery date (*n* = 2,422).

### Variables

MAX includes up to seven diagnosis codes per hospitalization. We determined values for covariates and outcomes using diagnostic and procedural codes from the ICD-9-CM and Physicians' Current Procedural Terminology codes. The dependent variables in this analysis were stillbirth (V27.1, V27.4, V27.7) and preterm birth (644.21).^21^

Independent variables in this analysis included the following demographic and clinical characteristics: maternal age, maternal race and/or ethnicity, year of birth, state of birth, Medicaid eligibility, location (rural residence), number of prenatal care visits, type of Medicaid coverage, substance use, comorbid conditions, and ART use. This information was collected from the Patient Personal Summary, Other Therapy, and Pharmacy files in MAX. We used three age categories (18–24, 25–34, and 35+) and treated age as a categorical variable. We used five race categories: White, Black, Hispanic, Other, and Unknown. Medicaid eligibility categories included poverty, Section 1931 Eligible (waiver allowing states to the raise income limit), Section 1115 Eligible (waiver allowing states to expand eligibility), Medically Needy (extends Medicaid eligibility to individuals with high medical expenses), and Other.

We dichotomized residence as rural versus nonrural using the 2010 Census ZCTA to Zip Code Crosswalk. Other than HIV, comorbid conditions were identified as previously described,^20^ and the number of individual conditions was classified as less than 2, 3, 4, or 5+ total conditions. We used two substance use categories: any substance use and tobacco use. We dichotomized Medicaid plan type into Fee for Service coverage (FFS) and Other (Managed Care, no insurance coverage, and Medicare dual eligible).

Following prior work, we defined an ART regimen as at least two prescription fills of at least three ingredient products (*e.g*., nucleoside reverse transcriptase inhibitors [NRTIs], nonnucleoside reverse transcriptase inhibitors [NNRTIs], PIs, or valid ART combinations).^19,22,23^ ART use was dichotomized as any ART versus none. The number of months of ART use in the year before delivery was categorized as 1, 3, 6, and 12 continuous months, with 12 months indicating that they were on ART preconception.

We counted only valid ART regimens (three ingredients) in this analysis; persons on suboptimal regimes were classified as not on ART. ART regimen type was classified as boosted PI-based, NRTI-based, NNRTI, and PI-based, and other (multiple classes). Integrase inhibitor regimens were rare and were included in the “other” category. We used patient admission date as a proxy for delivery date, and this delivery date was used to determine the type of Medicaid coverage (*e.g*., FFS, Medicaid Managed Care, Medicare dual eligible, or no insurance coverage) at time of delivery.

### Analyses

We compared sociodemographic and clinical characteristics of women using ART before delivery versus not and by category of duration of ART using chi-square tests. We next estimated two multivariate logistic regression models for stillbirth and preterm birth outcomes. The first regression analysis examined the impact of any ART use (vs. no ART use). We used propensity score-based inverse probability weights to adjust for differences between ART users and nonusers.^24–26^ The second regression analysis examined the impact of the number of months of ART use in the year before delivery using a similar approach.

In reporting the outcomes of these multivariable logistic regression models, we present marginal effects (MEs), or the change in probability when independent variable increases by one unit, rather than odds ratios.^27^ For example, using total live births among WWH on ART as the denominator, the probability of stillbirth and preterm birth were calculated at each level of ART months to determine if there were changes in the probability of the outcomes. Both models adjusted for age group, race, state, year, Medicaid eligibility, number of prenatal visits, comorbid conditions, substance use, and type of Medicaid coverage.

We do not show state coefficients in the tables because state Medicaid programs are very different from each other, and our focus is not on state differences; however, we do show models with state coefficients in an Supplementary Appendix. Additionally, balance and box plots for the propensity score matching are in the Supplementary Appendix. All statistical analyses were performed using SAS^®^ version 9.4 (SAS Institute, Cary, NC) and Stata^®^ 17.0 distributed by StataCorp LLC 2021. This study, and the waiver of informed consent, was reviewed and approved by the Brown University Institutional Review Board.

## Results

### Comparing mothers with and without ART

The final cohort included 23,755 unique women and 30,656 deliveries. Enrollee characteristics for mothers who did and did not initiate ART before delivery are shown in Table 1. Of 30,565 deliveries, 262 (0.9%) resulted in a stillbirth and 2,430 (8.0%) in a preterm birth. The proportion of stillbirth or preterm births between ART users and nonusers was not statistically significantly different. Compared to non-ART users, ART users were slightly older (46.1% vs. 47.1%, respectively, in the 25–34-year age range)—although there are no statistical differences between the three age groups, more often Black (34.4% vs. 32.2%), located in a rural area, were more likely to be on ART (7.7% vs. 6.7), had more prenatal care visits (30.3% vs. 17.6% with 7+ visits), more often used tobacco (16.8% vs. 15.1%), and more often had FFS Medicaid (62.4% and 59.1%, all *p* < 0.001).

**Table 1.** Descriptive Statistics, any Antiretroviral Therapy Use Before Delivery

	No ART	Any ART	*p*
*n* = 30,656	20,091	10,565	
Stillbirth	163 (0.8%)	99 (0.9%)	0.26
Preterm birth	1,598 (8.0%)	832 (7.9%)	0.81
Age groups			
18–24	7,972 (39.7%)	4,135 (39.1%)	0.29
25–34	9,270 (46.1%)	4,972 (47.1%)	
35+	2,849 (14.2%)	1,458 (13.8%)	
Race/ethnicity			
White	6,092 (30.3%)	3,230 (30.6%)	<0.001
Black	6,469 (32.2%)	3,633 (34.4%)	
Hispanic	4,883 (24.3%)	2,295 (21.7%)	
Other	2,020 (10.1%)	1,078 (10.2%)	
Unknown	627 (3.1%)	329 (3.1%)	
State			
CA	1,431 (7.1%)	601 (5.7%)	<0.001
FL	2,293 (11.4%)	1,501 (14.2%)	
GA	1,399 (7.0%)	920 (8.7%)	
IL	1,843 (9.2%)	821 (7.8%)	
LA	994 (4.9%)	463 (4.4%)	
MA	319 (1.6%)	166 (1.6%)	
MD	432 (2.2%)	328 (3.1%)	
NC	1,303 (6.5%)	757 (7.2%)	
NJ	247 (1.2%)	84 (0.8%)	
NY	4,804 (23.9%)	2,525 (23.9%)	
OH	533 (2.7%)	276 (2.6%)	
PA	415 (2.1%)	198 (1.9%)	
TX	3,511 (17.5%)	1,661 (15.7%)	
VA	567 (2.8%)	264 (2.5%)	
Years			
2001–2004	3,711 (18.5%)	2,919 (27.6%)	<0.001
2005–2008	6,782 (33.8%)	3,314 (31.4%)	
2009–2012	9,598 (47.8%)	4,332 (41.0%)	
Comorbid condition			
0 CC	2,101 (10.5%)	70 (0.7%)	<0.001
1 CC	4,259 (21.2%)	2,121 (20.1%)	
2 CC	2,015 (10.0%)	1,258 (11.9%)	
3 CC	2,283 (11.4%)	1,360 (12.9%)	
4 CC	1,744 (8.7%)	972 (9.2%)	
5+ CC	7,689 (38.3%)	4,784 (45.3%)	
Medicaid eligibility			
Poverty	6,278 (31.2%)	3,417 (32.3%)	0.032
Section 1931 eligible	5,682 (28.3%)	3,020 (28.6%)	
Section 1115 eligible	1,886 (9.4%)	937 (8.9%)	
Medically needy	2,018 (10.0%)	1,096 (10.4%)	
Other/unknown	4,227 (21.0%)	2,095 (19.8%)	
Location			
Rurality	1,343 (6.7%)	810 (7.7%)	0.001
Prenatal visits			
No visits	9,336 (46.5%)	3,267 (30.9%)	<0.001
1–3 visits	4,723 (23.5%)	2,449 (23.2%)	
4–6 visits	2,499 (12.4%)	1,664 (15.8%)	
7+ visits	3,533 (17.6%)	3,185 (30.1%)	
Medicaid coverage			
Other	8,218 (40.9%)	3,968 (37.6%)	<0.001
FFS	11,873 (59.1%)	6,597 (62.4%)	
ART regimen			
Boosted PI based		3,679 (34.8%)	<0.001
NRTI/NNRTI based		1,834 (17.4%)	
PI based		2,149 (20.3%)	
Multiple/other		2,903 (27.5%)	
Substance use			
Any substance use	5,088 (25.3%)	2,666 (25.2%)	0.86
Tobacco	3,023 (15.0%)	1,780 (16.8%)	<0.001

ART, antiretroviral therapy; CC, comorbid condition; FFS, fee for service; NRTI, nucleoside reverse transcriptase inhibitor; NNRTI, nonnucleoside reverse transcriptase inhibitor; PI, protease inhibitor.

### Comparing mothers by ART duration

A total of 10,656 deliveries out of 30,656 (34.6%) had any record of ART use (Tables 1 and 2). There were no statistically significant differences between the categories of months on ART for either stillbirth (*p* > 0.49) or premature birth (*p* > 0.68). Longer durations of ART use were observed for mothers with five or more comorbid conditions (31.2%, 34.2%, 39.4%, and 56.9% for 1, 3, 6, and 12 months of ART, respectively), mothers with seven or more prenatal visits (21.6%, 26.9%, 33.6%, and 34.3% for 1, 3, 6, and 12 months of ART, respectively), and those on a boosted PI-based ART regimen (34.1%, 36.9%, 42.1%, and 50.6% for 1, 3, 6, and 12 months of ART, respectively).

**Table 2.** Descriptive Statistics by Months on Antiretroviral Therapy Before Delivery

	1 Month	3 Months	6 Months	12 Months	*p*
*n* = 10,565	1,320	3,296	3,875	2,074	
Stillbirth	15 (1.1%)	35 (1.1%)	32 (0.8%)	17 (0.8%)	0.58
Preterm birth	97 (7.3%)	264 (8.0%)	294 (7.6%)	177 (8.5%)	0.52
Age groups					
18–24	551 (41.7%)	1,309 (39.7%)	1,557 (40.2%)	718 (34.6%)	<0.001
25–34	595 (45.1%)	1,558 (47.3%)	1,799 (46.4%)	1,020 (49.2%)	
35+	174 (13.2%)	429 (13.0%)	519 (13.4%)	336 (16.2%)	
Race/ethnicity					
White	388 (29.4%)	983 (29.8%)	1,194 (30.8%)	665 (32.1%)	<0.001
Black	437 (33.1%)	1,171 (35.5%)	1,359 (35.1%)	666 (32.1%)	
Hispanic	285 (21.6%)	697 (21.1%)	880 (22.7%)	433 (20.9%)	
Other	162 (12.3%)	353 (10.7%)	332 (8.6%)	231 (11.1%)	
Unknown	48 (3.6%)	92 (2.8%)	110 (2.8%)	79 (3.8%)	
State					
CA	64 (4.8%)	187 (5.7%)	229 (5.9%)	121 (5.8%)	<0.001
FL	221 (16.7%)	450 (13.7%)	573 (14.8%)	257 (12.4%)	
GA	87 (6.6%)	311 (9.4%)	348 (9.0%)	174 (8.4%)	
IL	91 (6.9%)	257 (7.8%)	292 (7.5%)	181 (8.7%)	
LA	60 (4.5%)	160 (4.9%)	166 (4.3%)	77 (3.7%)	
MA	16 (1.2%)	42 (1.3%)	65 (1.7%)	43 (2.1%)	
MD	41 (3.1%)	114 (3.5%)	122 (3.1%)	51 (2.5%)	
NC	92 (7.0%)	216 (6.6%)	290 (7.5%)	159 (7.7%)	
NJ	11 (0.8%)	28 (0.8%)	31 (0.8%)	14 (0.7%)	
NY	368 (27.9%)	764 (23.2%)	843 (21.8%)	550 (26.5%)	
OH	23 (1.7%)	89 (2.7%)	101 (2.6%)	63 (3.0%)	
PA	21 (1.6%)	63 (1.9%)	77 (2.0%)	37 (1.8%)	
TX	191 (14.5%)	513 (15.6%)	651 (16.8%)	306 (14.8%)	
VA	34 (2.6%)	102 (3.1%)	87 (2.2%)	41 (2.0%)	
Years					
2001–2004	397 (30.1%)	921 (27.9%)	1,090 (28.1%)	511 (24.6%)	<0.001
2005–2008	407 (30.8%)	1,097 (33.3%)	1,209 (31.2%)	601 (29.0%)	
2009–2012	516 (39.1%)	1,278 (38.8%)	1,576 (40.7%)	962 (46.4%)	
Comorbid condition					
≥2	586 (44.4%)	1,425 (43.2%)	1,398 (36.1%)	415 (20.0%)	<0.001
3 CC	191 (14.5%)	434 (13.2%)	554 (14.3%)	236 (11.4%)	
4 CC	131 (9.9%)	310 (9.4%)	398 (10.3%)	243 (11.7%)	
5+ CC	412 (31.2%)	1,127 (34.2%)	1,525 (39.4%)	1,180 (56.9%)	
Medicaid eligibility					
Poverty	415 (31.4%)	1,070 (32.5%)	1,274 (32.9%)	658 (31.7%)	0.007
Section 1931 eligible	391 (29.6%)	969 (29.4%)	1,105 (28.5%)	555 (26.8%)	
Section 1115 eligible	137 (10.4%)	301 (9.1%)	322 (8.3%)	177 (8.5%)	
Medically needy	140 (10.6%)	347 (10.5%)	405 (10.5%)	204 (9.8%)	
Other/unknown	237 (18.0%)	609 (18.5%)	769 (19.8%)	480 (23.1%)	
Location					
Rurality	96 (7.3%)	237 (7.2%)	350 (9.0%)	127 (6.1%)	<0.001
Prenatal visits					
No visits	470 (35.6%)	1,066 (32.3%)	1,164 (30.0%)	567 (27.3%)	<0.001
1–3 visits	372 (28.2%)	815 (24.7%)	797 (20.6%)	465 (22.4%)	
4–6 visits	193 (14.6%)	530 (16.1%)	611 (15.8%)	330 (15.9%)	
7+ visits	285 (21.6%)	885 (26.9%)	1,303 (33.6%)	712 (34.3%)	
Medicaid coverage					
Other	497 (37.7%)	1,278 (38.8%)	1,399 (36.1%)	794 (38.3%)	0.11
FFS	823 (62.3%)	2,018 (61.2%)	2,476 (63.9%)	1,280 (61.7%)	
ART regimen					
Boosted PI based	324 (24.5%)	926 (28.1%)	1,457 (37.6%)	972 (46.9%)	<0.001
NRTI/NNRTI based	298 (22.6%)	655 (19.9%)	631 (16.3%)	250 (12.1%)	
PI based	218 (16.5%)	639 (19.4%)	932 (24.1%)	360 (17.4%)	
Multiple/other	480 (36.4%)	1076 (32.6%)	855 (22.1%)	492 (23.7%)	
Substance use					
Any substance use	313 (23.7%)	786 (23.8%)	928 (23.9%)	639 (30.8%)	<0.001
Tobacco	171 (13.0%)	521 (15.8%)	641 (16.5%)	447 (21.6%)	<0.001

Descriptive, patient-level characteristics of Medicaid beneficiaries who gave birth between 2001 and 2012. This table illustrates the comparison of cumulative ART use 1, 3, 6, and 12 months before the delivery date.

### Stillbirth and preterm birth, any ART use

For mothers who initiated any ART before delivery, the probability for stillbirth (0.06%, 95% confidence interval [CI]: −0.17 to 0.28) or preterm birth (−0.12%, 95% CI: −0.79 to 0.56) was not statistically significant (Table 3). Black race was strongly associated with both outcomes (stillbirth: 0.80%, 95% CI: 0.45 to 1.15 and preterm birth: 4.19%, 95% CI: 3.29 to 5.08). Section 1931 Medicaid Eligibility was negatively associated with stillbirth (−0.49%, 95% CI: −0.82 to −0.16), as was an increased number of prenatal visits for preterm birth (−1.03%, 95% CI: −1.95 to −0.11 and −1.26%, 95% CI: −2.20 to −0.32). The same model, with state MEs, is shown in the Supplementary Appendix Table 1.

**Table 3.** Multivariable Logistic Regression, by Any Antiretroviral Therapy

***n*** = 30,656	Stillbirth	Preterm birth
Any ART	0.06%	−0.12%
Age group (Ref.: 17–24)	0.00%	0.00%
25–34	0.03%	0.08%
35+	0.14%	1.07%
Race/ethnicity (Ref.: White)		
Black	0.80%^*^	4.19%^*^
Hispanic	−0.20%	−0.12%
Other	0.13%	2.36%^*^
Unknown	0.38%	0.90%
Year (Ref.: 2001–2004)		
2005–2008	−0.03%	0.13%
2009–2012	−0.25%	−0.33%
Comorbid conditions (Ref.: ≤2)		
3 CC	0.10%	0.06%
4 CC	0.31%	0.77%
5+ CC	−0.13%	1.25%^**^
Medicaid eligibility (Ref.: Poverty)		
Section 1931 eligible	−0.49%^**^	0.15%
Section 1115 eligible	−0.06%	0.84%
Medically needy	−0.34%	−0.59%
Other/unknown	0.23%	0.38%
Location		
Rurality	0.25%	0.86%
Prenatal visits (Ref.: 0 visits)		
1–3 visits	0.25%	−1.03%^***^
4–6 visits	0.26%	−0.97%
7+ visits	0.19%	−1.26%^**^
Coverage (Ref.: Other)		
FFS	0.15%	0.27%
Substance use		
Any substance use	0.14%	0.45%
Tobacco	0.06%	0.44%

State is included in the model but not represented in the table, see Supplementary Appendix for state coefficients.

^*^ *p* < 0.001, ^**^p < 0.01, ^***^p < 0.05.

### Stillbirth and preterm birth, by months on ART and regimen type

After adjustment, the ME of ART duration was not statistically significant for either outcome (Table 4). When compared to 12 months of use, the percent difference in probability for individuals who had 1, 3, and 6 months of use of ART before delivery was 0.26% (95% CI: −0.51 to 1.03), 0.26% (95% CI: −0.35 to 0.88), and 0.05% (95% CI: −0.52 to 0.62) for stillbirth, respectively (Table 4). ART regimen type was not significantly associated with either outcome. Black race was strongly associated with both stillbirth (0.84%, 95% CI: 0.18 to 1.50) and preterm birth (3.76%, 95% CI: 2.13 to 5.40) when compared to White race. Section 1931 Medicaid Eligibility was associated with a decreased odds of stillbirth (−0.62%, 95% CI: −1.16 to −0.09).

**Table 4.** Multivariable Logistic Regression, by Months on Antiretroviral Therapy

***n*** = 10,565	Stillbirth	Preterm birth
Months on ART (Ref.: 12 Months)		
1 Months	0.26%	−0.39%
3 Months	0.26%	−0.37%
6 Months	0.05%	−0.69%
Age group (Ref.: 17–24)		
25–34	−0.11%	−0.29%
35+	−0.10%	0.59%
Race/ethnicity (Ref.: White)		
Black	0.84%^*^	3.76%^***^
Hispanic	−0.21%	0.39%
Other	0.53%	1.11%
Unknown	0.03%	−0.35%
Year (Ref.: 2001–2004)		
2005–2008	−0.05%	0.11%
2009–2012	−0.57%	0.25%
Comorbid conditions (Ref.: ≤2)		
3 CC	0.41%	0.37%
4 CC	0.86%	1.72%
5+ CC	0.12%	1.25%
Medicaid eligibility (Ref.: Poverty)		
Section 1931 eligible	−0.62%^*^	−1.29%
Section 1115 eligible	−0.22%	−0.94%
Medically needy	0.34%	0.30%
Other/unknown	0.77%	0.62%
Location		
Rurality	0.37%	−0.82%
Prenatal visits (Ref.: 0 Visits)		
1–3 visits	0.59%	−0.91%
4–6 visits	0.41%	−1.98%^*^
7+ visits	0.28%	−1.26%
Medicaid coverage (Ref.: Other)		
FFS	0.03%	0.79%
ART regimen (Ref.: NRTI/NNRTI based)		
Boosted PI based	−0.43%	−0.93%
PI based	−0.02%	0.23%
Other	−0.14%	−0.43%
Substance use		
Any substance use	−0.19%	−0.68%
Tobacco use	−0.11%	1.06%

State is included in the model but not represented in the table, see Supplementary Appendix for state coefficients.

^*^ *p* < 0.05, ^***^p < 0.001.

## Discussion

There are four main findings from this research. During the study period, only 34.5% of women who became pregnant and delivered a liveborn or, stillborn infant initiated ART before delivery. Second, neither ART use (vs. nonuse) nor duration of use among ART users was significantly associated with birth outcomes. Third, neither PI nor boosted PI regimens were more strongly associated with stillbirth or preterm birth than NRTI- and NNRTI-based regimens. Fourth, Black race was a strong, consistent predictor of adverse birth outcomes.

A recent analysis of all births in the United States reported rates of stillbirth and premature birth of 0.6% and 10.8%, respectively,^28^ which differ from our reported rates among this specific Medicaid population (0.9% and 7.9%). However, even with this, the rates of ART use within our study population were low. Low rates of ART use can result from multiple factors: (1) limited or nonregular engagement with primary care prepregnancy; (2) inadequate prenatal care, including routine HIV testing; (3) low rates of screening and testing of high-risk women; (4) stigma and additional barriers introduced by socioeconomic status; and (5) during the study period, ART treatment for individuals who were not pregnant was typically started either when their CD4 count reached a certain threshold or if they became pregnant.

Consequently, numerous women may not have begun treatment until after they had become pregnant. Our data cannot distinguish among these explanations. Nonetheless, our findings suggest potential issues in quality of care for pregnant WWH in the Medicaid in the United States.

Our analysis did not find significant differences in rates of stillbirth or preterm birth by ART use (vs. none), months of ART use (among ART users), or regimen type. There are a small handful of papers from the United States, and several from Europe,^29,30^ that examine adverse birth outcomes in WWH who use ART compared with nonusers. Among 488 pregnancies, Venkatesh et al. found that the risk of preterm birth only increased for pregnancies greater that 37 weeks.^14^

Two studies by Tuomala et al. found no association between ART use and birth outcomes.^10,11^ Studies by other teams that used data from single sites also showed no associations between ART and outcomes,^15^ similar to those assessing the Pediatric Spectrum of HIV Disease Cohort^16^ and deliveries in British Columbia.^31^ Even with differences between Europe and the United States—especially in terms of the health care system and access to care—our findings for stillbirth and preterm birth are similar to these other studies and extend the generalizability of these findings because we examined a large, population-based sample of low-income, Medicaid beneficiaries over a 12-year period.

We examined if there was an association between ART duration in the year before delivery and adverse birth outcomes and found no significant association. Albert et al. found that the cumulative amount of ART was associated with a lower rate of preterm birth in unadjusted analyses, but the effect disappeared in adjusted analyses, indicating that a longer duration of ART implies that the delivery is closer to term.^31^ Several articles have examined the issue of ART use in early versus late pregnancy, with varying results^11,17,29,30^; however, these studies primarily used single-site samples, or small cohorts. In our large sample from the United States comparing women on preconception ART use, it is important to note that there was a small increase in duration of ART after 2006, and increased ART use by women in our study over time. However, we did not find any significant trends either for duration of ART or for ART use at the time of conception.

Perhaps the biggest controversy in this area has been whether safety profiles vary with different ART regimens, with PI-based ART being the most often implicated regimen. Some studies suggest that adverse birth outcomes might be seen with combination ART,^32,33^ or with PI-based or boosted PI-based ART,^17,34–36^ The PROMISE trial, a randomized, controlled study that enrolled patients from 14 sites in seven non-U.S. countries, showed tenofovir-based ART, compared with zidovudine-based ART, was associated with higher rates of very preterm delivery and early infant death.^32^

A recent randomized control trial suggests that dolutegravir-based regimes have better virologic efficacy than an efavirenz-based regimen, and that a regimen of dolutegravir, emtricitabine, and tenofovir alafenamide fumarate has the lowest frequency of composite adverse birth outcomes and neonatal deaths (compared with the other two regimens tested).^37^ While ongoing assessments of the potential adverse effects of different ART regimens on birth outcomes are needed, particularly as new regimens are developed, the fact that our large, national study of Medicaid beneficiaries found no differences in rates of stillbirth or preterm birth by regimen type (PI-based, boosted PI-based, NRTI/NNRTI-based, and other) should be reassuring to pregnant women in the United States who are deciding about ART use.

After adjusting for social factors, we find that while Black women in our study used ART more frequently than other race/ethnicity groups, race/ethnicity was still the strongest predictor of stillbirth and preterm birth in our study. One implication of this finding is methodological. Studies of birth outcomes for WWH in the United States that do not (or cannot because of data limitations) control for race/ethnicity should be viewed with skepticism. More importantly, it is essential that these disparities in birth outcomes for Black women be eliminated. Barriers to timely and appropriate HIV care remain a barrier for Black persons with HIV across the treatment cascade,^38^ but these disparities are of course not limited to WWH.^39–41^

As a primary source of coverage for pregnant WWH, and particularly covering more than two-thirds of births among Black women, Medicaid can play a key role in helping to improve maternal health and reducing racial disparities in the long term. We advocate social and structural interventions that increase access to acceptable and culturally relevant care for disproportionately affected women of color to help close these racial/ethnic HIV disease–related access and care gaps, consistent with National HIV/AIDS Strategy goals.^42^

Our study has several limitations. First, we do not have data for all months of pregnancy for all women. Many study subjects qualified for Medicaid coverage because of pregnancy, which means we may have limited data on care in the months of pregnancy before coverage started and medication use. We also do not have data on potential care received from sources other than Medicaid, such as free care from a Federally Qualified Health Center or through a care site funded by the Ryan White Care Act. Hence, there is a potential for inaccuracies when determining the duration and initiation of ART.

Second, because we have only administrative claims data, we are unable to determine gestational age. This is an important limitation because (1) length of ART use is tied to length of gestation, which is used to define preterm birth; (2) the appropriate denominator for stillbirth is pregnancies at a given gestational age (*i.e*., fetuses at risk), and there are implications of using live births as the denominator instead^43,44^; and (3) associations between women on preconception ART versus postconception ART are prone to selection bias (*i.e*., confounding factors related to the selection of preconception ART that may influence results). Third, we do not have information on such potential confounders as nutrition, housing status, and social support. Fourth, claims data do not include information regarding early pregnancy loss or potentially important HIV-related laboratory data such as CD4 cell counts and viral loads.

Fifth, although we examined outcomes of over 30,000 pregnancies, stillbirth is still an uncommon birth outcome, and the 95% CIs around the odds ratios for stillbirth are somewhat broad. Sixth, understanding that stillbirth and preterm birth can reoccur, we chose to focus on total number of pregnancies rather than the total number of unique women to provide a more comprehensive picture of the study population, to fully examine the relationship between duration of ART treatment and adverse birth outcomes, increase statistical power and precision, and the highlight the relevance for clinical and public health implications in assessing the overall impact of ART on birth outcomes.

Seventh, while we chose to focus on pregnancy outcomes using inpatient claims, we recognize that the approach cannot capture early pregnancy losses that do not result in hospitalization. Eighth, these findings may not be generalizable to other states that we did not study, or to other parts of the world that have different systems of health care than the United States.

Finally, we do not believe the fact that we present data from before 2012 is a significant limitation. We show no differences in child outcomes in either the analysis of ART use or the analysis of the duration of ART use with the regimens in use at that time. ART regimes have improved in the intervening decade in ways that only make them safer to use. That is, population-level analyses of child health outcomes for pregnancies in Medicaid that use more current ART regimens would be less likely, not more likely, to show adverse birth outcomes. Had we found statistically significant difference using the data from the pre-2012 period, then it would have indeed been important to determine whether these differences were reduced with newer regimens. But given our null findings, we do not believe the fact that showing data from the period after 2012 is a serious limitation to the questions we ask about the relationship of ART use to rates of stillbirth and prematurity. That said, we hope that rates of ART use in pregnant persons have improved in the last decade, and that the racial disparities we show have diminished. More recent data are of course required to address these and other issues related to birth outcomes in Medicaid.

This study has several strengths. First, our observational study presents a relatively large sample of WWH (*n* = 30,656) and WWH on ART (*n* = 10,656) and assesses individuals enrolled in the Medicaid program over a 12-year period. Because the 14 states sampled have ∼75% of the cases of HIV in the United States, we believe that our findings are nationally generalizable for Medicaid. Second, the MAX database includes inpatient, outpatient, and prescription medication claims, ensuring considerable homogeneity in our ability to identify and code patient-level factors and treatment regimens for those on ART. 

Third, while we were not able to control for such factors as CD4 cell counts and viral loads, we were able to control for important potential confounders such as race/ethnicity, comorbid conditions, prenatal care, substance use, and smoking. Fourth, the way in which we coded for ART use means that we eliminated antiquated ART regimes such as single- and two-drug regimens. Fifth, we used propensity scores to adjust for differences between those who received ART and those who did not and to adjust for differences in ART duration. Sixth, and perhaps most importantly, we studied a population-based cohort of pregnancies in WWH. This study design avoids the selection effects necessarily seen in randomized trials, which often have restrictive entry criteria, and cohort studies, which are often based at academic medical centers and can suffer from participation bias.

In conclusion, among pregnant WWH in the United States covered by Medicaid between 2001 and 2012, neither ART use, the number of months of ART use, nor ART regimen type was associated with stillbirth or preterm birth. These results should be reassuring to both pregnant women and health care providers who are concerned that the advantages that ART offers to maternal health and vertical HIV transmission need to be balanced against potential risks for adverse birth outcomes. Our findings support Perinatal Guidelines^45^ and suggest that any such risks are small. We highlight that any ART treatment is better than no ART, both in terms of vertical transmission and improved outcomes for both the mother and the infant. Finally, the disparities in child health outcomes that we demonstrate for Black mothers are very concerning. Efforts to address the linked problems of disparities in maternal and child outcomes for Black mothers are urgently needed.

## Supplementary Material

Supplemental data

## Abbreviations Used

ART: antiretroviral therapy
CC: comorbid condition
CI: confidence interval
CMS: Centers for Medicare and Medicaid Services
FFS: fee for service
ICD-9-CM: International Classification of Diseases, Ninth Revision, Clinical Modification
MAX: Medicaid Analytic eXtract
ME: marginal effect
NRTI: nucleoside reverse transcriptase inhibitor
NNRTI: non-nucleoside reverse transcriptase inhibitor
PI: protease inhibitor
WWH: women living with HIV
