# Supplementary material for: Antiretroviral Therapy Use Was Not Associated with Stillbirth or Preterm Birth in an Analysis of U.S. Medicaid Pregnancies to Persons with HIV
Source: Womens Health Rep (New Rochelle). 2023 Aug 25;4(1):438–47. doi: 10.1089/whr.2023.0040 (PMC10457643; doi:10.1089/whr.2023.0040)
Supplement: Supplemental data [file Suppl_Appendix.docx]

**Appendix Tables:**

**Appendix Table 1: Multivariable Logistic Regression, Any ART**

***This is TABLE 3 with state included***

| N=30,656 | Stillbirth | 99% CI | Preterm Birth | 99% CI |
| --- | --- | --- | --- | --- |
|  |  |  |  |  |
| Any ART | 1.07 | (0.82 - 1.40) | 0.98 | (0.90 - 1.08) |
| Age Group (ref: 17-24) |  |  |  |  |
| 25-34 | 1.04 | (0.76 - 1.42) | 1.01 | (0.91 - 1.12) |
| 35+ | 1.17 | (0.76 - 1.81) | 1.15 | (0.99 - 1.34) |
| Race/Ethnicity (ref: White) |  |  |  |  |
| Black | 2.27** | (1.59 - 3.24) | 1.76** | (1.55 - 1.98) |
| Hispanic | 0.69 | (0.44 - 1.09) | 0.98 | (0.83 - 1.15) |
| Other | 1.20 | (0.67 - 2.15) | 1.42** | (1.18 - 1.70) |
| Unknown | 1.61 | (0.79 - 3.29) | 1.16 | (0.85 - 1.57) |
| State (ref: NY) |  |  |  |  |
| CA | 0.93 | (0.45 - 1.93) | 0.40** | (0.28 - 0.57) |
| FL | 0.71 | (0.41 - 1.20) | 0.82* | (0.68 - 0.98) |
| GA | 0.55 | (0.29 - 1.01) | 1.14 | (0.92 - 1.41) |
| IL | 1.06 | (0.60 - 1.86) | 1.07 | (0.88 - 1.31) |
| LA | 0.71 | (0.35 - 1.47) | 0.80 | (0.62 - 1.04) |
| MA | 1.05 | (0.42 - 2.63) | 0.53* | (0.33 - 0.86) |
| MD | 0.18 | (0.02 - 1.32) | 1.12 | (0.85 - 1.47) |
| NC | 1.13 | (0.61 - 2.10) | 1.11 | (0.90 - 1.36) |
| NJ | 1.42 | (0.52 - 3.91) | 0.82 | (0.52 - 1.30) |
| OH | 0.76 | (0.33 - 1.74) | 0.87 | (0.60 - 1.26) |
| PA | 0.99 | (0.36 - 2.73) | 1.26 | (0.92 - 1.74) |
| TX | 0.89 | (0.53 - 1.48) | 1.32** | (1.10 - 1.60) |
| VA | 1.35 | (0.64 - 2.86) | 1.24 | (0.92 - 1.67) |
| Year (ref: 2001-2004) |  |  |  |  |
| 2005-2008 | 0.97 | (0.69 - 1.36) | 1.02 | (0.90 - 1.15) |
| 2009-2012 | 0.74 | (0.50 - 1.09) | 0.95 | (0.84 - 1.09) |
| Comorbid Conditions (ref: <=2) |  |  |  |  |
| 3 CC | 1.11 | (0.72 - 1.72) | 1.01 | (0.86 - 1.18) |
| 4 CC | 1.37 | (0.88 - 2.15) | 1.12 | (0.94 - 1.32) |
| 5+ CC | 0.85 | (0.60 - 1.21) | 1.19** | (1.05 - 1.35) |
| Medicaid Eligibility (ref: Poverty) |  |  |  |  |
| Section 1931 Eligible | 0.51** | (0.32 - 0.80) | 1.02 | (0.89 - 1.17) |
| Section 1115 Eligible | 0.94 | (0.56 - 1.57) | 1.12 | (0.93 - 1.36) |
| Medically Needy | 0.66 | (0.36 - 1.18) | 0.92 | (0.75 - 1.12) |
| Other/Unknown | 1.23 | (0.86 - 1.75) | 1.05 | (0.91 - 1.21) |
| Location |  |  |  |  |
| Rurality | 1.31 | (0.80 - 2.14) | 1.12 | (0.94 - 1.34) |
| Prenatal Visits (ref: 0 Visits) |  |  |  |  |
| 1-3 Visits | 1.34 | (0.94 - 1.91) | 0.87* | (0.76 - 0.99) |
| 4-6 Visits | 1.36 | (0.88 - 2.10) | 0.88 | (0.75 - 1.02) |
| 7+ Visits | 1.27 | (0.87 - 1.85) | 0.84** | (0.74 - 0.96) |
| Coverage (ref: Other) |  |  |  |  |
| FFS | 1.21 | (0.89 - 1.63) | 1.04 | (0.94 - 1.15) |
| Substance Use |  |  |  |  |
| Any Substance Use | 1.18 | (0.85 - 1.64) | 1.06 | (0.94 - 1.21) |
| Tobacco | 1.08 | (0.75 - 1.54) | 1.06 | (0.93 - 1.22) |

** p<0.01, * p<0.05

**Appendix Table 2: Multivariable Logistic Regression, by Months on ART**

***This is TABLE 4 with state included***

| N=10,565 | Stillbirth | 99% CI | Preterm Birth | 99% CI |
| --- | --- | --- | --- | --- |
| Months on ART (ref: 12 Months) |  |  |  |  |
| 1 Months | 1.37 | (0.61 - 3.05) | 0.89 | (0.67 - 1.18) |
| 3 Months | 1.35 | (0.68 - 2.69) | 0.93 | (0.74 - 1.16) |
| 6 Months | 1.12 | (0.56 - 2.23) | 0.89 | (0.71 - 1.10) |
| Age Group (ref: 17-24) |  |  |  |  |
| 25-34 | 0.91 | (0.53 - 1.54) | 0.96 | (0.79 - 1.16) |
| 35+ | 0.96 | (0.45 - 2.09) | 1.08 | (0.83 - 1.40) |
| Race/Ethnicity (ref: White) |  |  |  |  |
| Black | 2.12* | (1.09 - 4.12) | 1.64** | (1.31 - 2.05) |
| Hispanic | 0.61 | (0.23 - 1.63) | 1.04 | (0.78 - 1.40) |
| Other | 1.67 | (0.58 - 4.82) | 1.20 | (0.86 - 1.67) |
| Unknown | 1.04 | (0.28 - 3.88) | 0.93 | (0.53 - 1.64) |
| State (ref: NY) |  |  |  |  |
| CA | 1.53 | (0.43 - 5.39) | 0.21** | (0.12 - 0.37) |
| FL | 0.78 | (0.33 - 1.83) | 0.76 | (0.56 - 1.02) |
| GA | 0.41 | (0.15 - 1.11) | 1.05 | (0.72 - 1.52) |
| IL | 0.70 | (0.25 - 1.92) | 0.94 | (0.66 - 1.33) |
| LA | 1.24 | (0.40 - 3.82) | 0.56* | (0.33 - 0.95) |
| MA | 0.42 | (0.05 - 3.25) | 0.27** | (0.10 - 0.70) |
| MD | 0.17 | (0.02 - 1.35) | 0.92 | (0.57 - 1.48) |
| NC | 1.33 | (0.55 - 3.22) | 0.85 | (0.59 - 1.21) |
| NJ | 1.92 | (0.25 - 14.49) | 0.46 | (0.15 - 1.46) |
| OH | 0.43 | (0.09 - 2.14) | 0.56 | (0.31 - 1.02) |
| PA | 1.51 | (0.38 - 6.07) | 0.91 | (0.50 - 1.66) |
| TX | 0.68 | (0.24 - 1.94) | 0.99 | (0.71 - 1.39) |
| VA | 1.57 | (0.55 - 4.44) | 1.54 | (0.91 - 2.62) |
| Year (ref: 2001-2004) |  |  |  |  |
| 2005-2008 | 1.00 | (0.57 - 1.76) | 1.02 | (0.81 - 1.27) |
| 2009-2012 | 0.55 | (0.28 - 1.05) | 1.01 | (0.79 - 1.29) |
| Comorbid Conditions (ref: <=2) |  |  |  |  |
| 3 CC | 1.41 | (0.61 - 3.24) | 1.04 | (0.79 - 1.36) |
| 4 CC | 2.16 | (0.98 - 4.79) | 1.23 | (0.89 - 1.68) |
| 5+ CC | 1.13 | (0.64 - 1.99) | 1.14 | (0.90 - 1.44) |
| Medicaid Eligibility (ref: Poverty) |  |  |  |  |
| Section 1931 Eligible | 0.40* | (0.19 - 0.84) | 0.83 | (0.65 - 1.07) |
| Section 1115 Eligible | 0.80 | (0.34 - 1.88) | 0.88 | (0.63 - 1.24) |
| Medically Needy | 1.28 | (0.53 - 3.12) | 1.00 | (0.70 - 1.43) |
| Other/Unknown | 1.65 | (0.86 - 3.16) | 1.06 | (0.82 - 1.38) |
| Location |  |  |  |  |
| Rurality | 1.45 | (0.70 - 2.97) | 0.90 | (0.66 - 1.24) |
| Prenatal Visits (ref: 0 Visits) |  |  |  |  |
| 1-3 Visits | 1.78 | (0.94 - 3.37) | 0.88 | (0.70 - 1.10) |
| 4-6 Visits | 1.61 | (0.72 - 3.61) | 0.79 | (0.59 - 1.05) |
| 7+ Visits | 1.36 | (0.67 - 2.75) | 0.84 | (0.67 - 1.07) |
| Medicaid Coverage (ref: Other) |  |  |  |  |
| FFS | 1.07 | (0.61 - 1.88) | 1.14 | (0.93 - 1.39) |
| ART Regimen (ref: NRTI Based) |  |  |  |  |
| Boosted PI Based | 1.02 | (0.52 – 2.01) | 0.97 | (0.68 - 1.38) |
| PI Based | 0.61 | (0.26 - 1.42) | 0.85 | (0.58- 1.24) |
| Other | 0.89 | (0.47 - 1.68) | 0.91 | (0.65 - 1.29) |
| Substance Use |  |  |  |  |
| Any Substance Use | 0.81 | (0.49 - 1.33) | 0.94 | (0.75 - 1.17) |
| Tobacco Use | 0.90 | (0.52 - 1.55) | 1.14 | (0.91 - 1.44) |

** p<0.01, * p<0.05

**Appendix Graph 1 and 2: Propensity of Any ART Use, Box and Balance Plots**

**Figure 1:**

**
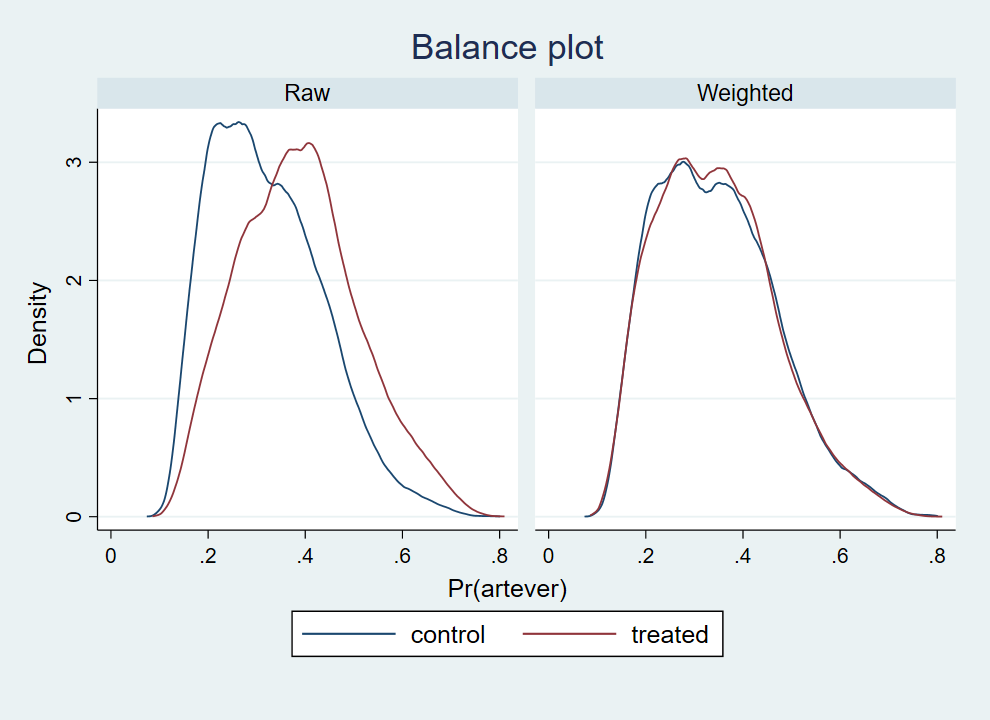
**

**Figure 2:**

**
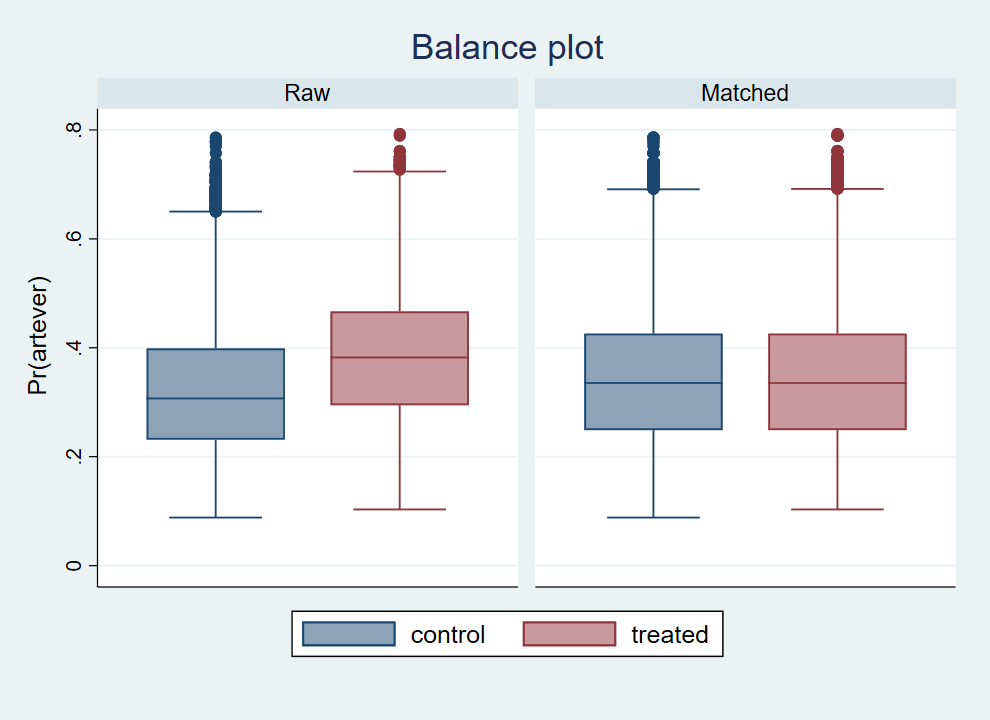
**
